# FAP in India: a first genetically proven case

**DOI:** 10.1186/1750-1172-10-S1-P20

**Published:** 2015-11-02

**Authors:** Daniel Pan, Jérôme Bouligand, Anne Guiochon-Mantel, David Adams

**Affiliations:** 1 Imperial College School of Medicine, Undergraduate student, SW7 2AZ, London, UK; 2 Publique Hôpitaux de Paris, Génétique moléculaire, Pharmacogénétique et Hormonologie, INSERM U693, Paris, France; 3 Publique Hôpitaux de Paris, French Reference Center for FAP (NNERF), INSERM U1191, Paris, France

## 

A 28 year old male with a background of abdominal tuberculosis living in Tekhand slum district of Delhi presented with two and half year history of recurrent loose stools following tingling sensation in the upper and lower limbs, as well as weakness in the lower limbs. He also reports erectile dysfunction and postural hypotension. He had lost weight : kilos. Upon examination the patient has lost all sensory perception in the lower limbs up to the upper thigh with severe wasting and dull lower limb reflexes. There was pes cavus and a high stepping gait.

Investigations revealed persistent circumferential thickening in the abdomen on CT. Sural nerve biopsy showed inflammation with demyelination and amyloidosis. There was a loss of parasympathic/sympathetic reactivity. Bone marrow biopsy revealed amyloid deposits compatible with secondary AA with increase in anti-LKM antibodies. Endoscopy revealed increased inflammation in the lamina propia. Familial amyloid polyneuropathy was suspected, and TTR Sanger sequencing revealed detection of a missense mutation of Val30Ala at a heterozygous state.

Transthyretin familial amyloid polyneuropathy (TTR-FAP) is a rare systemic disease due to endoneurial amyloid deposit, and is one of the most severe disabling hereditary peripheral neuropathies in adults. It has been described clinicopathologically in 1952 by Corino Andrade in north of Portugal and genetically in 1984 by Maria Joao Saraiva. TTR-FAP has long been considered an endemic disease in Portugal, Sweden and Japan, although sporadic cases have also been reported in Europe initially in France and the UK. There is genetic heterogeneity in TTR-FAP, with Val30Met being the most frequent genotype in the world. Val30Ala is a rare mutation, which have previously been described in the Chinese population.

In this manuscript, we document to our knowledge the first genetically proven case of FAP in India, a populated country of more than 1.2 billion inhabitants, and a detailed description of the patient case, as well as family investigations are provided. The patient is awaiting for anti-amyloid treatment.

Written informed consent for publication of their clinical details and/or clinical images was obtained from the patient /parent/guardian/ relative of the patient.

